# Supplementary material for: Organ aging signatures in the plasma proteome track health and disease
Source: Nature. 2023 Dec 6;624(7990):164–72. doi: 10.1038/s41586-023-06802-1 (PMC10700136; doi:10.1038/s41586-023-06802-1)
Supplement: Supplementary file 1 — Supplementary Discussion [file 41586_2023_6802_MOESM1_ESM.pdf]

nature portfolio

https://doi.org/10.1038/s41586-023-06802-1

Supplementary information

Organ aging signatures in the plasma proteome track health and disease

In the format provided by the authors and unedited

Organ aging signatures in the plasma proteome track health and disease

Hamilton Oh[1,2,3,#], Jarod Rutledge[2,3,4,#], Daniel Nachun[5], Róbert Pálovics[2,3,6], Olamide Abiose[3,6], Patricia Moran-Losada[2,3,6], Divya Channappa[2,3,6], Deniz Yagmur Urey[2,7], Kate Kim[2,3,6], Yun Ju Sung[8,9], Lihua Wang[8,9], Jigyasha Timsina[8,9], Dan Western[8,9,10], Menghan Liu[8,9], Pat Kohlfeld[8,9], John Budde[8,9], Edward N. Wilson[3,6], Yann Guen[6], Taylor M. Maurer[7], Michael Haney[2,3,6], Andrew C. Yang[11,12], Zihuai He[6], Michael D. Greicius[6], Katrin I. Andreasson[3,6], Sanish Sathyan[13], Erica F. Weiss[14], Sofiya Milman[13], Nir Barzilai[13], Carlos Cruchaga[8,9], Anthony Wagner[3], Elizabeth Mormino[6], Benoit Lehallier[6], Victor W. Henderson[3,6,15], Frank M. Longo[3,6], Stephen B. Montgomery[6,16], Tony Wyss-Coray[2,3,6,*]

1 Graduate Program in Stem Cell and Regenerative Medicine, Stanford University, Stanford, California, USA.
2 The Phil and Penny Knight Initiative for Brain Resilience, Stanford University, Stanford, California, USA.
3 Wu Tsai Neurosciences Institute, Stanford University, Stanford, CA, USA.
4 Graduate Program in Genetics, Stanford University, Stanford, California, USA.
5 Department of Pathology, Stanford University School of Medicine, Stanford, California, USA.
6 Department of Neurology and Neurological Sciences, Stanford University School of Medicine, Stanford, California, USA.
7 Department of Bioengineering, Stanford University School of Engineering, Stanford, CA, USA
8 Department of Psychiatry, Washington University in St Louis, St Louis, Missouri, USA
9 NeuroGenomics and Informatics Center, Washington University School of Medicine, St. Louis, MO, USA
10 Division of Biology and Biomedical Sciences, Washington University School of Medicine, St. Louis, MO, USA
11 Department of Anatomy, University of California San Francisco, San Francisco, CA, USA.
12 Bakar Aging Research Institute, University of California San Francisco, San Francisco, CA, USA.
13 Departments of Medicine and Genetics, Institute for Aging Research, Albert Einstein College of Medicine, Bronx, New York, USA.
14 Department of Neurology, Montefiore Medical Center, Bronx, New York, USA.
15 Department of Epidemiology and Population Health, Stanford University, Stanford, CA, USA.
16 Department of Genetics, Stanford University School of Medicine, Stanford, California, USA.

# these authors contributed equally
* correspondence to twc@stanford.edu

Table of Contents

A. Identification of putative organ-derived plasma proteins……………………………………………1

B. Non-linear associations between organ age gaps and mortality risk..……………………………1-2

C. Relationships between blood biochemistry markers and organ aging……………………………2-3

D. Relationship between CognitionBrain age gap and brain volume.………………………………...3-4

E. Literature review of highly weighted brain aging proteins...………………………………...............4-5

F. Complete study references....……………......................……………………………...............……5-9

Supplementary Discussion

A. Identification of putative organ-derived plasma proteins

We used the Gene Tissue Expression Atlas (GTEx) human tissue bulk RNA-seq database[16] to identify organ-specific genes and plasma proteins. We determined organ-specificity based on a 4-fold cutoff in bulk RNA-seq data for three main reasons:

1. Determining tissue specificity based on a 4-fold increase in RNA-seq expression ("tissue-enriched") from GTEx and other databases is a well-accepted approach, established by the Human Protein Atlas (HPA) in multiple studies[81–83]. The HPA's tissue-enriched gene sets are widely trusted and are provided in NCBI, GeneCards, and enrichment analysis tools such as gprofiler[71]. We used the same metric but with the updated, more deeply sequenced GTEx RNA-seq dataset and with a more generalizable framework for tissue->organ mapping (Supplementary Table 2).
2. We considered determining organ-specificity based on tissue protein levels from a human tissue proteomics atlas (Jiang et al)[84]; however, we opted not to because organ protein levels may be misleading in regard to determining the original organ source of the protein. Specifically, a protein may be present in an organ because it was trafficked there after being synthesized by another organ and secreted into the plasma. Albumin and complement proteins are not enriched at the protein level in the liver even though they are synthesized there, and there are proteins which are synthesized in the hypothalamus that are enriched in the pituitary because they are stored there before release[84]. Generally, discordance between protein and RNA levels are interpreted as a result of protein trafficking/export/secretion, while enrichment at the RNA level is recognized as the tissue of origin for protein synthesis[81,82,84,85]. It may also be true that proteins which are present at the protein level in an organ but are not synthesized there also contain important information about said organ. We believe this idea of cross-organ communication in aging is an exciting area for future study. For the current manuscript, our goal was to determine the putative organ source of plasma proteins to infer organ age.
3. RNA-seq data contains nearly full coverage of the genome, while proteomics data has much lower coverage. In Jiang et al, only 6320 proteins were detected in >50% of samples, and these are heavily biased towards abundant proteins, which are detectable by mass spectrometry. The percentage of these mappable to the SomaScan plasma proteomics assay is even lower. Determining organ-specificity based on RNA-seq data increased our coverage of the mappable organ-specific plasma proteome.

B. Non-linear associations between organ age gaps and mortality risk.

To better understand potential non-linear associations between age gaps and disease risk, we performed a binned age gap versus mortality risk analysis in the LonGenity cohort (Supplementary Figure 4). Specifically, we binned individuals into different age gap groups:

- Bin –2 (–2.5 < age gap < –1.5)
- Bin –1 (–1.5 < age gap < –0.5)
- Bin 0 (–0.5 < age gap < +0.5)
- Bin +1 (+0.5 < age gap < +1.5)
- Bin +2 (+1.5 < age gap < +2.5)
- Bin +3 (+2.5 < age gap < +3.5)

- Bin –3 and other more extreme bins were removed due to low sample size.

We then compared every non-zero group with the zero group (denoting the non-zero group as 1 and the zero group as 0) for changes in mortality risk. We did this analysis for each of the aging models. We did not adjust for multiple comparisons because the assumptions were not met: each statistical test is done in a different subset of individuals, and tests for different bins in the same organ are generally correlated.

Interestingly, the association between the age gap and mortality risk was non-linear for some organs, such as the heart, brain, pancreas, kidney. The relationship with the heart age gap seems to be U-shaped where both high (+1, +2, +3) and extremely low heart age gaps (–2) are associated with increased mortality risk. The kidney age gap was also interesting in that it was not associated with mortality risk when looking at the whole age gap distribution (Fig. 2j), but the +3 age gap group was positively associated with mortality, suggesting the "extreme agers" framework may be more useful for certain organs and traits. Other organs, including the organismal, adipose, artery, and immune, show a more linear relationship with mortality risk. Whether these nonlinear dynamics also exist for other aging biomarkers, such as methylation clocks, is unknown. This analysis points to a need for additional studies on the relationship between extreme aging and disease risk.

C. Relationships between blood biochemistry markers and organ aging.

While a full analysis of all clinical biochemistry markers is challenging, there are a number of additional interesting relationships in the data.

- BUN: Kidney, adipose, brain, immune, and muscle age gaps are significantly positively associated with BUN, artery age gap is significantly negatively associated. The strongest association is with the kidney age gap. While BUN is not specific, it is often considered a marker of kidney function clinically.
- AST: Kidney, heart, and artery age gaps are positively significantly associated with AST, while brain is significantly negatively associated. AST variation within the normal range is difficult to interpret clinically. Abnormally high AST is often a sign of liver or heart disease, and moderately high AST is most often noted as a sign of elevated cardiovascular risk in middle aged and elderly populations.
- ALT: The brain, control, liver, intestine, kidney, organismal, and pancreas age gaps are significantly negatively associated with ALT, while the kidney age gap is significantly positively associated with ALT. PhenoAge gap is positive but not significant. As discussed in the text, this is yet another U-shaped aging biomarker. Low ALT in the elderly is associated with increased frailty and reduced survival and has been previously suggested as a biomarker of aging[86]. Abnormally high ALT can be a marker of acute liver damage, although it is also produced by other tissues and is not specific.
- Albumin: The immune, heart, liver, organismal, control, and PhenoAge gaps are significantly negatively associated with albumin levels. The strongest association is with the liver age gap. Albumin is produced by the liver, although it is not detected by the SomaScan assay so it is not a protein in the liver aging model. Clinically, lower albumin could be considered as a sign of worse health, and it can be low in a number of liver, kidney, and digestive diseases as well as in malnutrition/undernutrition.
- Plasma glucose is significantly positively associated with PhenoAge age gap and kidney age gap, while intestine and liver age gap are significantly negatively associated. The strongest association is with PhenoAge, which is unsurprising since plasma glucose is the highest weighted input biomarker in the PhenoAge model. Both kidney and intestine

age gap are positively associated with diabetes incidence but have differential associations with plasma glucose. This further supports the hypothesis that different organ models could be measuring different aspects of aging, in this case metabolic aging. Insulin resistance, glucose response, and glucose levels are all known to degrade with age, but insulin levels and glucose response have been noted to change more dramatically than fasting blood glucose level[87].

There are many biomarkers of health which have a nonlinear relationship to aging outcomes, and in the elderly many relationships between biomarkers and health/mortality/frailty reverse direction compared to young and middle-aged adults. The distribution and mean age of the population that an aging model is trained on will thus impact associations with traits. This is not frequently discussed or accounted for in models of molecular aging.

Such a case is illustrated by diastolic blood pressure, where the strongest association was with heart aging (adjusted Pearson r=-0.18, q=2.62e-10). Nine organ age gaps (adipose, brain, control, heart, intestine, kidney, liver, muscle, organismal, pancreas) were significantly associated with decreases in diastolic blood pressure, while the opposite association was seen with the PhenoAge age gap (Supplementary Fig. 5a, Supplementary Table 14). Diastolic blood pressure was one of many traits with a U-shaped relationship to aging outcomes (Supplementary Fig. 5b). While high blood pressure in young and middle-aged adults is indicative of cardiometabolic dysfunction, in the elderly low blood pressure is common and more strongly associated with mortality and frailty[88–90], though high blood pressure is also detrimental[91]. The differences between PhenoAge and the organ age models could be due to differences in the age distribution of the underlying training cohorts for the models. Our models were trained in the KADRC, which has a greater proportion of elderly individuals, while PhenoAge was trained in NHANES, which has a greater proportion of young individuals.

This kind of U-shaped relationship with age and aging outcomes is quite common and is also seen with BMI[92]. Prospective studies in older adults have shown that while obesity slightly increases mortality and cardiovascular disease risk, the highest risk groups are those with a BMI under 23. Interestingly, the intestine and pancreas age gaps show a negative association with BMI and obesity but a positive association with mortality risk, while the kidney age gap shows a positive association with BMI, suggesting that the full picture of organ health in aging and disease may be more complex than currently understood.

D. Relationship between CognitionBrain age gap and brain volume.

To further examine the relationship between the CognitionBrain age model and brain aging, we tested associations between CognitionBrain age gap and changes in brain volume. We used plasma-matched brain MRI data from 469 individuals in the Stanford-ADRC and SAMS cohorts to assess the relationship between the CognitionBrain age gap and brain region-specific volumes (Extended Data Fig. 7c, Supplementary Table 22). 39 out of 65 (60%) associations were significant after multiple hypothesis correction. The most significant associations were negative associations with the superior frontal cortex (adjusted r=-0.20, q=8.49e-5), hippocampus (adjusted r=-0.21, q=1.36e-4), and total cortex (adjusted r=-0.20, q=1.39e-4), whereby individuals with smaller brain region volumes appeared older based on their CognitionBrain age gaps. We also found a negative association with the AD signature region (adjusted r=-0.16, q=3.61e-3), a composite measure of the parahippocampal gyrus, entorhinal cortex, inferior parietal lobes, hippocampus, and precuneus[93].

We then compared our plasma proteomics-based brain age to two MRI brain aging clocks. Based on its established publication record, we started with the BARACUS model[78], a linear support vector machine based aging clock trained on brain MRI-based volumetric data from 1,166 cognitively normal individuals aged 20-80. However, when assessing predicted versus chronological age correlation, we noticed an odd technical artifact: the predicted age had a ceiling

near 75, even for individuals with chronological age above 90. Looking more closely at the original publication, we found the same issue of an upper ceiling, and also a lower ceiling, to predicted age. This leads us to believe that the BARACUS algorithm cannot accommodate all ages in our cohort.

Due to this technical limitation of BARACUS, we also assessed brainageR[14], a Gaussian Processes based aging clock trained on brain MRI-based volumetric data from n=3,377 cognitively healthy individuals aged 18-92, and which has shown better performance than BARACUS in other studies[94]. The CognitionBrain age gap was positively correlated with the brainageR age gap (r=0.16, p=7.51e-4) (Extended Data Fig. 6h), but not as strongly as the correlation between CognitionBrain age gap and individual brain volumes (ie. hippocampus: adjusted r=-0.21, q=1.36e-4). This is likely due to the fact that BARACUS and brainageR do not take into account total intracranial volume and thus capture more noise.

E. Literature review of highly weighted brain aging proteins.

Protein	Reference	Title	Organism
CPLX1	Yu, et. al., *JAMA Psychiatry* (2020)	Cortical Proteins Associated with Cognitive Resilience in Community-Dwelling Older Persons	Human
CPLX1	Glynn, et. al., *Human Molecular Genetics* (2005)	Profound ataxia in complexin I knockout mice masks a complex phenotype that includes exploratory and habituation deficits	Mouse
CPLX1,CPLX2	Tannenberg, et. al., *Neurochemistry International* (2006)	Selective loss of synaptic proteins in Alzheimer's disease: Evidence for an increased severity with *APOE* ε4	Human
CPLX2	Begemann, et al. *Arch. Gen. Psychiatry* (2010)	Modification of Cognitive Performance in Schizophrenia by Complexin 2 Gene Polymorphisms	Human
CPLX2	Glynn, et. al., *Human Molecular Genetics* (2003)	Complexin II is essential for normal neurological function in mice	Mouse
CPLX2	Li, et. al., *Oxidative Medicine and Cellular Longevity* (2020)	Proteomic Profile of Mouse Brain Aging Contributions to Mitochondrial Dysfunction, DNA Oxidative Damage, Loss of Neurotrophic Factor, and Synaptic and Ribosomal Proteins	Mouse
FGF4	Konijnenberg, et. al., *Alzheimer's Research & Therapy* (2020)	APOE ε4 genotype-dependent cerebrospinal fluid proteomic signatures in Alzheimer's disease	Human
FGF4	Kosaka, et. al., *Federation of American Societies for Experimental Biology* (2006)	FGF-4 regulates neural progenitor cell proliferation and neuronal differentiation	Mouse
LANCL1	Drummond, et. al., *Brain* (2020)	Phosphorylated tau interactome in the human Alzheimer's disease brain	Human
LANCL1	Huang, Chen, and Peng, et. al., *Developmental Cell* (2014)	Developmental and Activity-Dependent Expression of LanCL1 Confers Antioxidant Activity Required for Neuronal Survival	Mouse
LANCL1	Tan and Chen, et. al., *Cell Death & Differentiation* (2020)	LanCL1 promotes motor neuron survival and extends the lifespan of amyotrophic lateral sclerosis mice	Mouse
NPTXR	Begcevic, et. al., *F1000Research* (2018)	Neuronal pentraxin receptor-1 is a new cerebrospinal fluid biomarker of Alzheimer's disease progression	Human
NPTXR	Pelkey, et. al. *Neuron* (2015)	Pentraxins Coordinate Excitatory Synapse Maturation and Circuit Integration of Parvalbumin Interneurons	Mouse
NRXN3	Hishimoto, et. al., *Alzheimer's Research & Therapy* (2019)	Neurexin 3 transmembrane and soluble isoform expression and splicing haplotype are associated with neuron inflammasome and Alzheimer's disease	Human
NRXN3	Zheng, et. al., *Medicine* (2018)	Low expression of aging-related NRXN3 is associated with Alzheimer disease	Human
NRXN3	Martinez-Mir, et. al., *J Alz Disease* (2013)	Genetic study of neurexin and neuroligin genes in Alzheimer's disease	Human
NRXN3	Aoto, et. al. *Nature Neuroscience* (2015)	Distinct circuit-dependent functions of presynaptic neurexin-3 at GABAergic and glutamatergic synapses	Mouse

Protein	Reference	Title	Organism
OLFM1	Nakaya, et. al., *Experimental Neurology* (2013)	Deletion in the N-terminal half of olfactomedin 1 modifies its interaction with synaptic proteins and causes brain dystrophy and abnormal behavior in mice	Mouse
OLFM1	Nakaya, et. al., *Journal of Biological Chemistry* (2012)	Olfactomedin 1 Interacts with the Nogo A Receptor Complex to Regulate Axon Growth	Mouse
STMN2	Theunissen, et. al., *Frontiers in Aging Neuroscience* (2021)	Novel STMN2 Variant Linked to Amyotrophic Lateral Sclerosis Risk and Clinical Phenotype	Human
STMN2	Krus, et. al., *Cell Reports* (2022)	Loss of Stathmin-2, a hallmark of TDP-43-associated ALS, causes motor neuropathy	Human
STMN2	San Juan and Nash, et. al., *Neuron* (2022)	Loss of mouse Stmn2 function causes motor neuropathy	Mouse
TNR	Wagner, et. al., *Genetics in Medicine* (2020)	Loss of TNR causes a nonprogressive neurodevelopmental disorder with spasticity and transient opisthotonus	Human
TNR	Dankovich, et. al., *Nature Communications* (2021)	Extracellular matrix remodeling through endocytosis and resurfacing of Tenascin-R	Mouse
TNR	Bauch and Faissner, *Cells* (2022)	The Extracellular Matrix Proteins Tenascin-C and Tenascin-R Retard Oligodendrocyte Precursor Maturation and Myelin Regeneration in a Cuprizone-Induced Long-Term Demyelination Animal Model	Mouse

F. Complete study references

1. Schaum, N. *et al.* Ageing hallmarks exhibit organ-specific temporal signatures. *Nature* **583**, 596–602 (2020).
2. Almanzar, N. *et al.* A single-cell transcriptomic atlas characterizes ageing tissues in the mouse. *Nature* **583**, 590–595 (2020).
3. Pálovics, R. *et al.* Molecular hallmarks of heterochronic parabiosis at single-cell resolution. *Nature* **603**, 309–314 (2022).
4. Zahn, J. M. *et al.* AGEMAP: A Gene Expression Database for Aging in Mice. *PLOS Genet.* **3**, e201 (2007).
5. Janelidze, S. *et al.* Plasma P-tau181 in Alzheimer's disease: relationship to other biomarkers, differential diagnosis, neuropathology and longitudinal progression to Alzheimer's dementia. *Nat. Med.* **26**, 379–386 (2020).
6. Hajat, C. & Stein, E. The global burden of multiple chronic conditions: A narrative review. *Prev. Med. Rep.* **12**, 284–293 (2018).
7. Kaeberlein Matt, Rabinovitch Peter S., & Martin George M. Healthy aging: The ultimate preventative medicine. *Science* **350**, 1191–1193 (2015).
8. Conboy, I. M. *et al.* Rejuvenation of aged progenitor cells by exposure to a young systemic environment. *Nature* **433**, 760–764 (2005).
9. Rutledge, J., Oh, H. & Wyss-Coray, T. Measuring biological age using omics data. *Nat. Rev. Genet.* 1–13 (2022) doi:10.1038/s41576-022-00511-7.
10. Putin, E. *et al.* Deep biomarkers of human aging: Application of deep neural networks to biomarker development. *Aging* **8**, 1021–1030 (2016).
11. Belsky, D. W. *et al.* Quantification of biological aging in young adults. *Proc. Natl. Acad. Sci.* **112**, E4104–E4110 (2015).
12. Levine, M. E. *et al.* An epigenetic biomarker of aging for lifespan and healthspan. *Aging* **10**, 573–591 (2018).
13. Tian, Y. E. *et al.* Heterogeneous aging across multiple organ systems and prediction of chronic disease and mortality. *Nat. Med.* **29**, 1221–1231 (2023).
14. Cole, J. H. *et al.* Brain age predicts mortality. *Mol. Psychiatry* **23**, 1385–1392 (2018).

15. Glorioso, C., Oh, S., Douillard, G. G. & Sibille, E. Brain Molecular Aging, Promotion of Neurological Disease and Modulation by Sirtuin5 Longevity Gene Polymorphism. *Neurobiol. Dis.* **41**, 279–290 (2011).
16. Consortium, T. Gte. The GTEx Consortium atlas of genetic regulatory effects across human tissues. *Science* **369**, 1318–1330 (2020).
17. Uhlén Mathias *et al.* Tissue-based map of the human proteome. *Science* **347**, 1260419 (2015).
18. Hannum, G. *et al.* Genome-wide Methylation Profiles Reveal Quantitative Views of Human Aging Rates. *Mol. Cell* **49**, 359–367 (2013).
19. Horvath, S. DNA methylation age of human tissues and cell types. *Genome Biol.* **14**, 3156 (2013).
20. Tanaka, T. *et al.* Plasma proteomic signature of age in healthy humans. *Aging Cell* **17**, e12799 (2018).
21. Lehallier, B. *et al.* Undulating changes in human plasma proteome profiles across the lifespan. *Nat. Med.* **25**, 1843–1850 (2019).
22. Sparks, M. A., Crowley, S. D., Gurley, S. B., Mirotsou, M. & Coffman, T. M. Classical Renin-Angiotensin System in Kidney Physiology. in *Comprehensive Physiology* 1201–1228 (John Wiley & Sons, Ltd, 2014). doi:10.1002/cphy.c130040.
23. Buchanan, S., Combet, E., Stenvinkel, P. & Shiels, P. G. Klotho, Aging, and the Failing Kidney. *Front. Endocrinol.* **11**, (2020).
24. Gudbjartsson, D. F. *et al.* Association of Variants at UMOD with Chronic Kidney Disease and Kidney Stones—Role of Age and Comorbid Diseases. *PLOS Genet.* **6**, e1001039 (2010).
25. Devuyst, O. & Pattaro, C. The UMOD Locus: Insights into the Pathogenesis and Prognosis of Kidney Disease. *J. Am. Soc. Nephrol.* **29**, 713 (2018).
26. Shrivastava, A., Haase, T., Zeller, T. & Schulte, C. Biomarkers for Heart Failure Prognosis: Proteins, Genetic Scores and Non-coding RNAs. *Front. Cardiovasc. Med.* **7**, (2020).
27. Ho, J. E. *et al.* Protein Biomarkers of Cardiovascular Disease and Mortality in the Community. *J. Am. Heart Assoc.* **7**, e008108 (2018).
28. Saberi, S. *et al.* Mavacamten Favorably Impacts Cardiac Structure in Obstructive Hypertrophic Cardiomyopathy. *Circulation* **143**, 606–608 (2021).
29. McCrory, C. *et al.* GrimAge Outperforms Other Epigenetic Clocks in the Prediction of Age-Related Clinical Phenotypes and All-Cause Mortality. *J. Gerontol. Ser. A* **76**, 741–749 (2021).
30. Bellenguez, C. *et al.* New insights into the genetic etiology of Alzheimer’s disease and related dementias. *Nat. Genet.* **54**, 412–436 (2022).
31. Yu, L. *et al.* Cortical Proteins Associated With Cognitive Resilience in Community-Dwelling Older Persons. *JAMA Psychiatry* **77**, 1172–1180 (2020).
32. Begemann, M. *et al.* Modification of Cognitive Performance in Schizophrenia by Complexin 2 Gene Polymorphisms. *Arch. Gen. Psychiatry* **67**, 879–888 (2010).
33. Hishimoto, A. *et al.* Neurexin 3 transmembrane and soluble isoform expression and splicing haplotype are associated with neuron inflammasome and Alzheimer’s disease. *Alzheimers Res. Ther.* **11**, 28 (2019).
34. Klim, J. R. *et al.* ALS-implicated protein TDP-43 sustains levels of STMN2, a mediator of motor neuron growth and repair. *Nat. Neurosci.* **22**, 167–179 (2019).
35. Nakaya, N., Sultana, A., Lee, H.-S. & Tomarev, S. I. Olfactomedin 1 Interacts with the Nogo A Receptor Complex to Regulate Axon Growth*. *J. Biol. Chem.* **287**, 37171–37184 (2012).

36. Yin, G. N., Lee, H. W., Cho, J.-Y. & Suk, K. Neuronal pentraxin receptor in cerebrospinal fluid as a potential biomarker for neurodegenerative diseases. *Brain Res.* **1265**, 158–170 (2009).
37. Bader, J. M. *et al.* Proteome profiling in cerebrospinal fluid reveals novel biomarkers of Alzheimer's disease. *Mol. Syst. Biol.* **16**, e9356 (2020).
38. Tan, H. *et al.* LanCL1 promotes motor neuron survival and extends the lifespan of amyotrophic lateral sclerosis mice. *Cell Death Differ.* **27**, 1369–1382 (2020).
39. Johnson, E. C. B. *et al.* Large-scale deep multi-layer analysis of Alzheimer's disease brain reveals strong proteomic disease-related changes not observed at the RNA level. *Nat. Neurosci.* **25**, 213–225 (2022).
40. Tang, W., Huang, Q., Wang, Y., Wang, Z.-Y. & Yao, Y.-Y. Assessment of CSF Aβ42 as an aid to discriminating Alzheimer's disease from other dementias and mild cognitive impairment: A meta-analysis of 50 studies. *J. Neurol. Sci.* **345**, 26–36 (2014).
41. THE TABULA SAPIENS CONSORTIUM. The Tabula Sapiens: A multiple-organ, single-cell transcriptomic atlas of humans. *Science* **376**, eabl4896 (2022).
42. Yang, A. C. *et al.* A human brain vascular atlas reveals diverse mediators of Alzheimer's risk. *Nature* **603**, 885–892 (2022).
43. Sengillo, J. D. *et al.* Deficiency in Mural Vascular Cells Coincides with Blood–Brain Barrier Disruption in Alzheimer's Disease. *Brain Pathol.* **23**, 303–310 (2013).
44. Nikolakopoulou, A. M. *et al.* Pericyte loss leads to circulatory failure and pleiotrophin depletion causing neuron loss. *Nat. Neurosci.* **22**, 1089–1098 (2019).
45. Callegari, A., Coons, M. L., Ricks, J. L., Rosenfeld, M. E. & Scatena, M. Increased Calcification in Osteoprotegerin-Deficient Smooth Muscle Cells: Dependence on Receptor Activator of NF-κB Ligand and Interleukin 6. *J. Vasc. Res.* **51**, 118–131 (2014).
46. Köhler, S. *et al.* The Human Phenotype Ontology in 2021. *Nucleic Acids Res.* **49**, D1207–D1217 (2021).
47. Qureshi, A. R. *et al.* Increased circulating sclerostin levels in end-stage renal disease predict biopsy-verified vascular medial calcification and coronary artery calcification. *Kidney Int.* **88**, 1356–1364 (2015).
48. Touw, W. A. *et al.* Association of Circulating Wnt Antagonists With Severe Abdominal Aortic Calcification in Elderly Women. *J. Endocr. Soc.* **1**, 26–38 (2017).
49. Lu, A. T. *et al.* DNA methylation GrimAge strongly predicts lifespan and healthspan. *Aging* **11**, 303–327 (2019).
50. Ganz, P. *et al.* Development and Validation of a Protein-Based Risk Score for Cardiovascular Outcomes Among Patients With Stable Coronary Heart Disease. *JAMA* **315**, 2532–2541 (2016).
51. Williams, S. A. *et al.* Plasma protein patterns as comprehensive indicators of health. *Nat. Med.* **25**, 1851–1857 (2019).
52. Gubbi, S. *et al.* Effect of Exceptional Parental Longevity and Lifestyle Factors on Prevalence of Cardiovascular Disease in Offspring. *Am. J. Cardiol.* **120**, 2170–2175 (2017).
53. Sathyan, S. *et al.* Plasma proteomic profile of age, health span, and all-cause mortality in older adults. *Aging Cell* **19**, e13250 (2020).
54. Wilson, E. N. *et al.* Performance of a fully-automated Lumipulse plasma phospho-tau181 assay for Alzheimer's disease. *Alzheimers Res. Ther.* **14**, 172 (2022).
55. Berg, L. *et al.* Clinicopathologic Studies in Cognitively Healthy Aging and Alzheimer Disease: Relation of Histologic Markers to Dementia Severity, Age, Sex, and Apolipoprotein E Genotype. *Arch. Neurol.* **55**, 326–335 (1998).
56. Morris, J. C. The Clinical Dementia Rating (CDR): Current version and scoring rules. *Neurology* **43**, 2412-a (1993).
57. Gold, L. *et al.* Aptamer-Based Multiplexed Proteomic Technology for Biomarker Discovery. *PLOS ONE* **5**, e15004 (2010).

58. SomaLogic. SOMAscan® v4 Data Standardization and File Specification Technical Note.
59. SomaLogic. SomaScan® v4 Data Standardization. (2020).
60. SomaLogic. Technical Specification: Adaptive Normalization Using Maximum Likelihood.
61. Candia, J., Daya, G. N., Tanaka, T., Ferrucci, L. & Walker, K. A. Assessment of variability in the plasma 7k SomaScan proteomics assay. *Sci. Rep.* **12**, 17147 (2022).
62. Katz, D. H. *et al.* Proteomic profiling platforms head to head: Leveraging genetics and clinical traits to compare aptamer- and antibody-based methods. *Sci. Adv.* **8**, eabm5164 (2022).
63. SomaSignal Tests - Products and Services. *SomaLogic* https://somalogic.com/somasignal-tests-for-research-use/.
64. Love, M. I., Huber, W. & Anders, S. Moderated estimation of fold change and dispersion for RNA-seq data with DESeq2. *Genome Biol.* **15**, 550 (2014).
65. Pedregosa, F. *et al.* Scikit-learn: Machine Learning in Python. *J. Mach. Learn. Res.* **12**, 2825–2830 (2011).
66. Seabold, S. & Perktold, J. Statsmodels: Econometric and Statistical Modeling with Python. in *Proceedings of the 9th Python in Science Conference* (eds. Walt, S. van der & Millman, J.) 92–96 (2010). doi:10.25080/Majora-92bf1922-011.
67. Levine, M. E. *et al.* An epigenetic biomarker of aging for lifespan and healthspan. *Aging* **10**, 573–591 (2018).
68. Viechtbauer, W. Conducting Meta-Analyses in R with the metafor Package. *J. Stat. Softw.* **36**, 1–48 (2010).
69. Davidson-Pilon, Cameron. (2022). lifelines, survival analysis in Python (v0.27.0). Zenodo. https://doi.org/10.5281/zenodo.6359609.
70. Breiman, L. Random Forests. *Mach. Learn.* **45**, 5–32 (2001).
71. Raudvere, U. *et al.* g:Profiler: a web server for functional enrichment analysis and conversions of gene lists (2019 update). *Nucleic Acids Res.* **47**, W191–W198 (2019).
72. Szklarczyk, D. *et al.* The STRING database in 2021: customizable protein–protein networks, and functional characterization of user-uploaded gene/measurement sets. *Nucleic Acids Res.* **49**, D605–D612 (2021).
73. Litviňuková, M. *et al.* Cells of the adult human heart. *Nature* **588**, 466–472 (2020).
74. Stewart Benjamin J. *et al.* Spatiotemporal immune zonation of the human kidney. *Science* **365**, 1461–1466 (2019).
75. Michael S. Haney *et al.* APOE4/4 is linked to damaging lipid droplets in Alzheimer’s microglia. *bioRxiv* 2023.07.21.549930 (2023) doi:10.1101/2023.07.21.549930.
76. Johnson, E. C. B. *et al.* Large-scale deep multi-layer analysis of Alzheimer’s disease brain reveals strong proteomic disease-related changes not observed at the RNA level. *Nat. Neurosci.* **25**, 213–225 (2022).
77. Fischl, B. FreeSurfer. *NeuroImage* **62**, 774–781 (2012).
78. Liem, F. *et al.* Predicting brain-age from multimodal imaging data captures cognitive impairment. *NeuroImage* **148**, 179–188 (2017).
79. Poplin, R. *et al.* Scaling accurate genetic variant discovery to tens of thousands of samples. 201178 Preprint at https://doi.org/10.1101/201178 (2018).
80. Chang, C. C. *et al.* Second-generation PLINK: rising to the challenge of larger and richer datasets. *GigaScience* **4**, s13742-015-0047–8 (2015).
81. Uhlén, M. *et al.* Tissue-based map of the human proteome. *Science* **347**, (2015).
82. Uhlén, M. *et al.* The human secretome. *Sci. Signal.* **12**, (2019).
83. Uhlen, M. *et al.* A genome-wide transcriptomic analysis of protein-coding genes in human blood cells. *Science* **366**, (2019).
84. Jiang, L. *et al.* A Quantitative Proteome Map of the Human Body. *Cell* **183**, 269-283.e19 (2020).

85. Liu, Y., Beyer, A. & Aebersold, R. On the Dependency of Cellular Protein Levels on mRNA Abundance. *Cell* **165**, 535–550 (2016).
86. Le Couteur, D. G. *et al.* The Association of Alanine Transaminase With Aging, Frailty, and Mortality. *J. Gerontol. A. Biol. Sci. Med. Sci.* **65A**, 712–717 (2010).
87. Bryhni, B., Arnesen, E. & Jenssen, T. G. Associations of age with serum insulin, proinsulin and the proinsulin-to-insulin ratio: a cross-sectional study. *BMC Endocr. Disord.* **10**, 21 (2010).
88. Protogerou, A. D. *et al.* Diastolic Blood Pressure and Mortality in the Elderly With Cardiovascular Disease. *Hypertension* **50**, 172–180 (2007).
89. Taylor, J. O. *et al.* Blood Pressure and Mortality Risk in the Elderly. *Am. J. Epidemiol.* **134**, 489–501 (1991).
90. Boshuizen, H. C., Izaks, G. J., Buuren, S. van & Ligthart, G. J. Blood pressure and mortality in elderly people aged 85 and older: community based study. *BMJ* **316**, 1780–1784 (1998).
91. Glynn, R. J. *et al.* Evidence for a positive linear relation between blood pressure and mortality in elderly people. *The Lancet* **345**, 825–829 (1995).
92. Ng, T. P. *et al.* Age-dependent relationships between body mass index and mortality: Singapore longitudinal ageing study. *PLOS ONE* **12**, e0180818 (2017).
93. Walker, K. A. *et al.* Large-scale plasma proteomic analysis identifies proteins and pathways associated with dementia risk. *Nat. Aging* **1**, 473–489 (2021).
94. Clausen, A. N. *et al.* Assessment of brain age in posttraumatic stress disorder: Findings from the ENIGMA PTSD and brain age working groups. *Brain Behav.* **12**, e2413 (2022).
