# Supplementary material for: Organ aging signatures in the plasma proteome track health and disease
Source: Nature. 2023 Dec 6;624(7990):164–72. doi: 10.1038/s41586-023-06802-1 (PMC10700136; doi:10.1038/s41586-023-06802-1)
Supplement: Supplementary file 2 — Reporting Summary [file 41586_2023_6802_MOESM2_ESM.pdf]

nature portfolio

Corresponding author(s): Tony Wyss-Coray

Last updated by author(s): Sep 22, 2023

Reporting Summary

Nature Portfolio wishes to improve the reproducibility of the work that we publish. This form provides structure for consistency and transparency in reporting. For further information on Nature Portfolio policies, see our Editorial Policies and the Editorial Policy Checklist.

Statistics

For all statistical analyses, confirm that the following items are present in the figure legend, table legend, main text, or Methods section.

n/a	Confirmed	
☐	☒	The exact sample size (n) for each experimental group/condition, given as a discrete number and unit of measurement
☐	☒	A statement on whether measurements were taken from distinct samples or whether the same sample was measured repeatedly
☐	☒	The statistical test(s) used AND whether they are one- or two-sided<br>*Only common tests should be described solely by name; describe more complex techniques in the Methods section.*
☐	☒	A description of all covariates tested
☐	☒	A description of any assumptions or corrections, such as tests of normality and adjustment for multiple comparisons
☐	☒	A full description of the statistical parameters including central tendency (e.g. means) or other basic estimates (e.g. regression coefficient) AND variation (e.g. standard deviation) or associated estimates of uncertainty (e.g. confidence intervals)
☐	☒	For null hypothesis testing, the test statistic (e.g. F, t, r) with confidence intervals, effect sizes, degrees of freedom and P value noted<br>*Give P values as exact values whenever suitable.*
☒	☐	For Bayesian analysis, information on the choice of priors and Markov chain Monte Carlo settings
☒	☐	For hierarchical and complex designs, identification of the appropriate level for tests and full reporting of outcomes
☐	☒	Estimates of effect sizes (e.g. Cohen's d, Pearson's r), indicating how they were calculated

Our web collection on statistics for biologists contains articles on many of the points above.

Software and code

Policy information about availability of computer code

Data collection	Plasma proteomics data were collected through Somalogic Inc's.

Data analysis

Python 3.9.12
gprofiler-official==1.0.0
lifelines==0.27.3
matplotlib==3.5.1
numpy==1.21.5
pandas==1.4.2
scanpy==1.9.1
scikit-learn==1.0.2
scikit-survival==0.17.2
scipy==1.10.0
seaborn==0.12.2
statsmodels==0.13.5

R 4.1.2
metafor==4.2.0
DESeq2=1.38.3

For manuscripts utilizing custom algorithms or software that are central to the research but not yet described in published literature, software must be made available to editors and reviewers. We strongly encourage code deposition in a community repository (e.g. GitHub). See the Nature Portfolio guidelines for submitting code & software for further information.

Data

Policy information about availability of data

All manuscripts must include a data availability statement. This statement should provide the following information, where applicable:

- Accession codes, unique identifiers, or web links for publicly available datasets
- A description of any restrictions on data availability
- For clinical datasets or third party data, please ensure that the statement adheres to our policy

Stanford-ADRC data are available upon reasonable request to the Stanford-ADRC data release committee, https://web.stanford.edu/group/adrc/cgi-bin/web-proj/datareq.php. All Stanford-ADRC data will be made publicly available after an embargo period at this link: https://twc-stanford.shinyapps.io/adrc/. SAMS data are available to qualified investigators upon request to principal investigators Beth Mormino (bmormino@stanford.edu) or Anthony Wagner (awagner@stanford.edu). Knight-ADRC data were generated by the lab of principal investigator Carlos Cruchaga (cruchagac@wustl.edu) and are available upon reasonable request to the NIAGADS (Study ID: ng00130), https://www.niagads.org/knight-adrc-collection. Data from the Covance and LonGenity cohorts can be accessed according to the policies described in the initial study publications51–53.

Pre-processed human heart73 and kidney74 scRNA-seq data were accessed from studies in the Human Cell Atlas. Pre-processed brain scRNA-seq data were accessed from Haney et. al. 202375. Pre-processed human brain vasculature scRNA-seq data were accessed from Yang et. al. 202242. Pre-processed human vasculature scRNA-seq data were accessed from Tabula Sapiens41. Differential expression statistics of proteins and RNA from Alzheimer's disease versus control brains were accessed from Johnson et. al. 202276.

Change with age information of ~5,000 SomaScan v4 plasma proteins across all 5 cohorts (Supplementary Fig. 8, Supplementary Table 25) are available in a public shiny app (https://twc-stanford.shinyapps.io/aging_plasma_proteome_v2/).

Research involving human participants, their data, or biological material

Policy information about studies with human participants or human data. See also policy information about sex, gender (identity/presentation), and sexual orientation and race, ethnicity and racism.

Reporting on sex and gender

"Models that included sex as a covariate and models trained separately on males and females showed similar age prediction performance on both sexes, so we controlled for sex to extend the generality of the findings and reduce analytic complexity (Supplementary Fig. 3 a-c)."

Reporting on race, ethnicity, or other socially relevant groupings

Biological differences between different ethnic/racial groups were not assessed in this study. Ethnicity distributions per cohort are available in Supplementary Table 1.

Population characteristics

Covance
Details of the Covance study have been previously published77. Briefly, Covance is a multi-site cross-sectional study of health across the lifespan collected at 5 hospital sites in the United States in 2008. 1028 subjects were included in analyses for this study. Cohort demographic characteristics are summarized in Supplementary Table 1. Exclusion criteria for the study included uncontrolled hypertension, self-reported treatment for a malignancy other than squamous cell or basal cell carcinoma of the skin in the last 2 years, self-reported pregnancy, self-reported chronic infection, autoimmune condition or other inflammatory condition, self-reported chronic kidney or liver disease, chronic heart failure or diagnosed with myocardial infarction in the last 3 months, self-reported diabetes (HbA1c>8% if known), self-reported acute bacterial or viral infection in the past 24 hours or a temperature > 38 C within 24 hours of enrollment, self-reported participation in any therapeutic study within 14 days prior of blood sampling, and taking more than 20 mg of prednisone or related drugs.

Clinical blood chemistry performed on the same samples, including a complete blood count and comprehensive metabolic panel, lipid panel, and liver function tests. Basic physical workup (blood pressure, pulse, respirations) was also collected.

Lifestyle information was also collected from all participants using a survey which asked about smoking, alcohol, exercise, habits, and frequency of consumption of different meats and vegetables.

LonGenity
Details of the LonGenity cohort have been previously published78,79. Briefly, LonGenity is an ongoing longitudinal study initiated in 2008 and designed to identify biological factors that contribute to healthy aging. The LonGenity study enrolls older adults of Ashkenazi Jewish descent with age 65–94 years at baseline. Approximately half of the cohort consists of offspring of parents with exceptional longevity, defined as having at least one parent who survived to 95 years of age. The other half of the cohort includes offspring of parents with usual survival, defined as not having a parental history of exceptional longevity. 962 subjects were included in analyses for this study. Cohort characteristics are summarized in Supplementary Table 1. LonGenity participants are thoroughly characterized demographically and phenotypically at annual visits that include collection of medical history and physical and detailed neurocognitive assessments (described in detail below). The LonGenity study was approved by the institutional review board (IRB) at the Albert Einstein College of Medicine.

Subjects in the LonGenity cohort underwent extensive cognitive examination. The Overall Cognition Composite score was determined by the relative performance of the subject in the Free and Cued Selective Reminding Test, WMS-R Logical Memory I, RBANS Figure Copy, RBANS Figure Recall, WAIS-III Digit Span, WAIS-III Digit Symbol Coding, Phonemic Fluency (FAS), Categorical Fluency, Trail Making Test A, and Trail Making Test B. For each task a standardized score (z) was calculated based on the population. The z for each task is then combined to create the Overall Cognition Composite.

Stanford Alzheimer's Disease Research Center (Stanford-ADRC)
Samples were acquired through the National Institute on Aging (NIA)-funded Stanford Alzheimer's Disease Research Center (Stanford-ADRC). The Stanford-ADRC cohort is a longitudinal observational study of clinical dementia subjects and age-sex-matched non-demented subjects. The collection of plasma was approved by the Institutional Review Board of Stanford University and written consent was obtained from all subjects. Blood collection and processing were done according to a rigorous standardized protocol to minimize variation associated with blood draw and blood processing. Briefly, about 10 cc whole blood was collected in a vacutainer EDTA tube (BD Vacutainer EDTA tube) and spun at 3000RPM for 10 mins to separate out plasma, leaving 1 cm of plasma above the buffy coat and taking care not to disturb the buffy coat to circumvent cell contamination. Plasma processing times averaged approximately one hour from the time of the blood draw to the time of freezing and storage. All blood draws were done in the morning to minimize the impact of circadian rhythm on protein concentrations. Plasma pTau-181 levels were measured using the fully-automated Lumipulse G 1200 platform (Fujirebio US, Inc, Malvern, PA) by experimenters blind to diagnostic information, as previously described42.

All healthy control participants were deemed cognitively unimpaired during a clinical consensus conference that included board-certified neurologists and neuropsychologists. Cognitively impaired subjects underwent Clinical Dementia Rating and standardized neurological and neuropsychological assessments to determine cognitive and diagnostic status, including procedures of the National Alzheimer's Coordinating Center (https://naccdata.org/). Cognitive status was determined in a clinical consensus conference that included neurologists and neuropsychologists. All participants were free from acute infectious diseases and in good physical condition. 412 subjects were included in analyses for this study. Cohort demographics and clinical diagnostic categories are summarized in Supplementary Table 1.

Stanford Aging Memory Study (SAMS)
SAMS is an ongoing longitudinal study of healthy aging. Blood collection and processing were done by the same team and using the same protocol as in Stanford-ADRC. Neurological and neuropsychological assessment were done by the same team and using the same protocol as in Stanford-ADRC. All SAMS participants had CDR=0 and neuropsychological test score within normal range; all SAMS participants were deemed cognitively unimpaired during a clinical consensus conference that included neurologists and neuropsychologists. 192 cognitively SAMS participants were included in the present study, and 11 were participants in both the Stanford-ADRC and SAMS study. Cohort demographics and clinical diagnostic categories are summarized in Supplementary Table 1.

Knight Alzheimer's Disease Research Center (Knight-ADRC)
The Knight ADRC (Knight-ADRC) cohort is an NIA-funded longitudinal observational study of clinical dementia subjects and age-matched controls. Research participants at the Knight-ADRC undergo longitudinal cognitive, neuropsychologic, imaging, and biomarker assessments including Clinical Dementia Rating (CDR). Among individuals with CSF and plasma data, AD cases corresponded to those with a diagnosis of dementia of the Alzheimer's type (DAT) using criteria equivalent to the National Institute of Neurological and Communication Disorders and Stroke-Alzheimer's Disease and Related Disorders Association for probable AD80 and AD severity was determined using the Clinical Dementia Rating (CDR®)81 at the time of lumbar puncture (for CSF samples) or blood draw (for plasma samples). Controls received the same assessment as the cases but were non-demented (CDR=0). Blood samples were collected in EDTA tubes (BD Vacutainer purple top) at the visit time, immediately centrifuged at 1500g for 10 minutes, aliquoted on 2D barcoded Micronic tubes (200ul per aliquot) and stored at − 80°C. The plasma was stored in monitored -80C freezer until it was pulled and sent to Somalogic for data generation. The Institutional Review Board of Washington University School of Medicine in St. Louis approved the study and research was performed in accordance with the approved protocols. 3075 participants were included in the present study. Cohort demographics and clinical diagnostic categories are summarized in Supplementary Table 1.

Recruitment

Participants were recruited through the NIA-funded Stanford Alzheimer's Disease Research Center and the Knight Alzheimer's Disease Research Center. Recruitment of healthy controls is biased towards significant others/partners/relatives of diseased patients, which may affect findings.

Ethics oversight

LonGenity cohort: Institutional Review Board of the Albert Einstein College of Medicine. Stanford-ADRC and SAMS cohorts: Institutional Review Board of Stanford University. Knight-ADRC cohort: Institutional Review Board of Washington University School of Medicine in St. Louis

Note that full information on the approval of the study protocol must also be provided in the manuscript.

Field-specific reporting

Please select the one below that is the best fit for your research. If you are not sure, read the appropriate sections before making your selection.

☒ Life sciences ☐ Behavioural & social sciences ☐ Ecological, evolutionary & environmental sciences

For a reference copy of the document with all sections, see nature.com/documents/nr-reporting-summary-flat.pdf

Life sciences study design

All studies must disclose on these points even when the disclosure is negative.

Sample size	No power analyses were used to predetermine sample sizes. However, sample sizes were informed by prior literature using similar experimental paradigms that yielded interpretable results and the lab's previous experience. For example in Lehallier et al. Nature Medicine 2019, we measured ~3,000 proteins from 4,263 individuals. Here we measure ~5,000 from 5,676 individuals.
Data exclusions	Samples flagged by Somalogic for quality control were excluded.
Replication	All plasma proteomic measurements were acquired from unique human samples.
Randomization	Samples from healthy individuals and individuals with neurodegenerative disease were age and sex matched.
Blinding	Training and testing of models were performed on completely independent datasets. Investigators responsible for plasma and data collection were blinded to patient demographics.

Reporting for specific materials, systems and methods

We require information from authors about some types of materials, experimental systems and methods used in many studies. Here, indicate whether each material, system or method listed is relevant to your study. If you are not sure if a list item applies to your research, read the appropriate section before selecting a response.

Materials & experimental systems

n/a	Involved in the study	
☒	☐	Antibodies
☒	☐	Eukaryotic cell lines
☒	☐	Palaeontology and archaeology
☒	☐	Animals and other organisms
☒	☐	Clinical data
☒	☐	Dual use research of concern
☒	☐	Plants

Methods

n/a	Involved in the study	
☒	☐	ChIP-seq
☒	☐	Flow cytometry
☐	☒	MRI-based neuroimaging

Magnetic resonance imaging

Experimental design

Design type	Resting state
Design specifications	NA
Behavioral performance measures	NA

Acquisition

Imaging type(s)	structural
Field strength	3T
Sequence & imaging parameters	Whole-brain MRI scans were collected from all subjects in the Stanford-ADRC and SAMS cohorts. All MRI data was collected at the Stanford Richard M. Lucas Center for Imaging. 271 subjects underwent MRI scanning on a 3T MRI scanner (GE Discovery MR750). T1-weighted SPGR scans were collected (TR/TE/TI=8.2/3.2/900ms, flip angle=9, 1x1x1mm) and used to define gray matter volumes. 134 subjects underwent MRI scanning on hybrid PET/MRI scanner (Signa 3 tesla, GE Healthcare). T1-weighted SPGR scan were collected (TR/TE/TI=7.7/3.1/400ms, flip angle=11, 1.2x1.1x1.1mm) and used to define gray matter volumes.
Area of acquisition	whole brain

Diffusion MRI ☐ Used ☒ Not used

Preprocessing

Preprocessing software	Region of interest (ROI) labeling was implemented using the FreeSurfer100 software package version 7 (http://surfer.nmr.mgh.harvard.edu). In brief, structural images were bias field corrected, intensity normalized, and skull stripped using a watershed algorithm. These images underwent a white matter-based segmentation, grey/white matter and pial surfaces were defined, and topology correction was applied to these reconstructed surfaces. Subcortical and cortical ROIs spanning the entire brain were defined in each subject's native space, using the aparc+aseg atlas in FreeSurfer.
Normalization	T1-w SPGR scans were normalized according to FreeSurfer's recon-all command
Normalization template	MNI305
Noise and artifact removal	NA
Volume censoring	NA

Statistical modeling & inference

Model type and settings	basic multivariate linear models were used to associate brain ROI volumes with plasma proteomic aging signatures, while accounting for age and sex.
Effect(s) tested	only brain ROI volumes were assessed.

Specify type of analysis: ☐ Whole brain ☒ ROI-based ☐ Both

Anatomical location(s)	Subcortical and cortical ROIs spanning the entire brain were defined in each subject's native space, using the aparc+aseg atlas in FreeSurfer.
Statistic type for inference (See Eklund et al. 2016)	*Specify voxel-wise or cluster-wise and report all relevant parameters for cluster-wise methods.*
Correction	*Describe the type of correction and how it is obtained for multiple comparisons (e.g. FWE, FDR, permutation or Monte Carlo).*

Models & analysis

n/a	Involved in the study
☒	☐ Functional and/or effective connectivity
☒	☐ Graph analysis
☐	☒ Multivariate modeling or predictive analysis

Multivariate modeling and predictive analysis	Using matched brain MRI and plasma proteomic data from n=541 samples in SAMS and Stanford-ADRC, we compared our plasma proteomic organ clocks with established brain MRI based-clocks, brainageR and BARACUS Brain-Age.<br><br>We used a pre-trained machine learning algorithm (https://github.com/james-cole/brainageR) and raw T1-weighted MRI scans to estimate brain age. This software uses SPM12 (https://www.fil.ion.ucl.ac.uk/spm/software/spm12/) to perform tissue segmentation and normalization of individual scans to Montreal Neurological Institute (MNI) template space. The software relies on a model that used Gaussian process regression to predict brain age on 3,777 participants from seven publicly available datasets (mean age = 40.1, range = 18-90 years). It applies the results of this training to predict brain age in any new T1-w data, utilizing the RNifti (version 1.4.5) and kernlab (version 0.9-32) packages within R version 4.2.

We also used another pre-trained algorithm, BARACUS (https://github.com/bids-apps/baracus; Liem et al. 2017), to estimate brain age from FreeSurfer version 5.3 processed T1-w scans. The vertex-wise cortical thickness and surface area values (transformed from subject space to fsaverage4 standard space), along with the subcortical volumetric statistics, were used as input to BARACUS's linear support vector machine model. This model was trained on 1,166 participants with no objective cognitive impairment (566 female, mean age = 59.1, range = 20-80 years). It returns a "stacked-anatomy" prediction among its results, which we used as the estimate of brain age for this method.
