# Supplementary material for: Organ aging signatures in the plasma proteome track health and disease
Source: Nature. 2023 Dec 6;624(7990):164–72. doi: 10.1038/s41586-023-06802-1 (PMC10700136; doi:10.1038/s41586-023-06802-1)
Supplement: Supplementary file 3 — Supplementary Figures 1–8. [file 41586_2023_6802_MOESM3_ESM.pdf]

Supplementary Figures

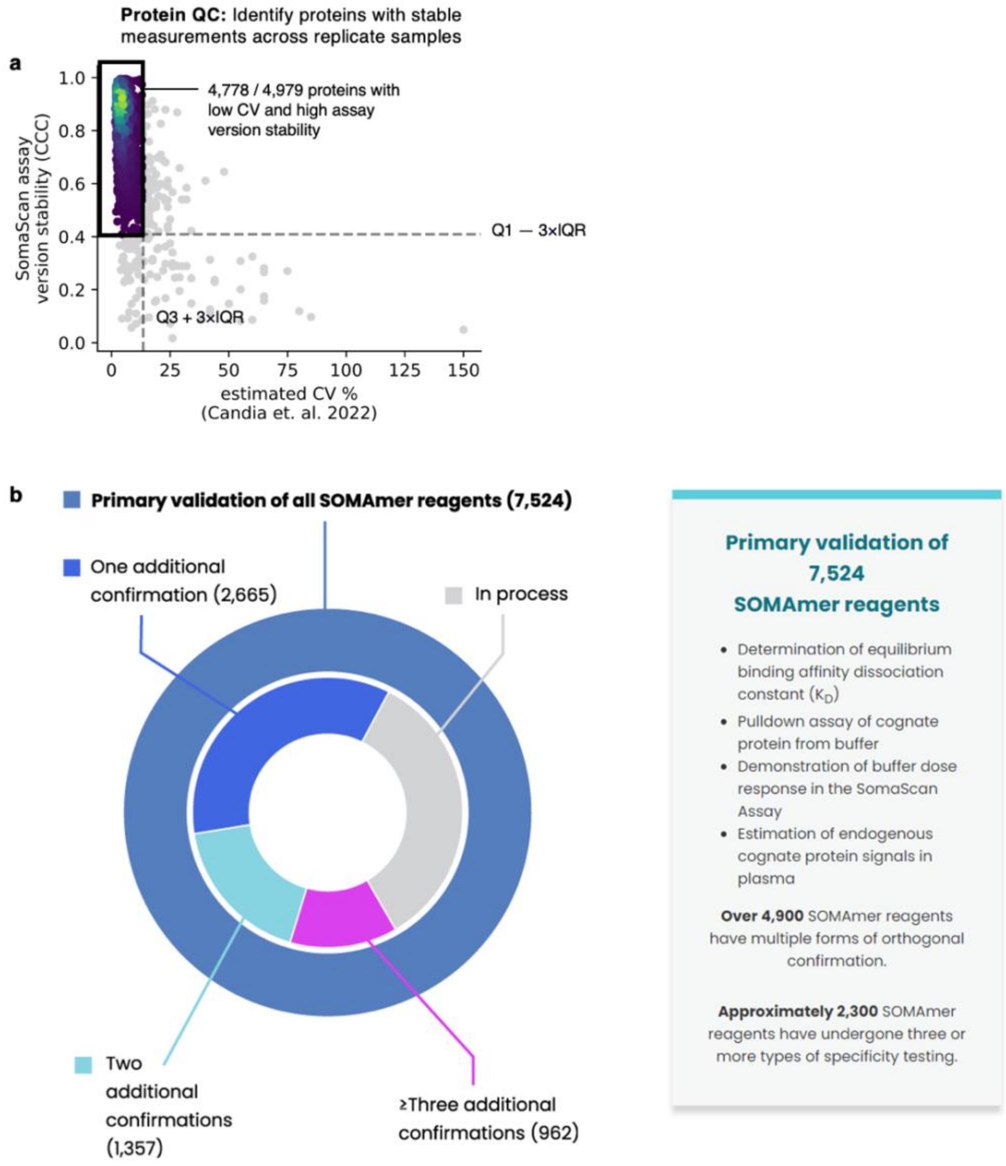

Supplementary Figure 1. Protein quality control

a, Replicate measurement reproducibility assessed using 1) Lin's concordance correlation coefficient (CCC) between replicate samples across SomaScan v4 and v4.1 assay versions (data provided by Somalogic) and 2) estimated coefficient of variation (CV) based on replicate samples in Candia, et. al. 2022. Proteins with high outlier values–- based on 3 times the interquartile range–- for these metrics were removed.

b, Somalogic's quality control pipeline for the SomaScan assay. All probes on the assay undergo rigorous primary validation of sensitivity and specificity to the target protein. Additional experimental validation (ie. mass-spec, antibody, cis-pQTL, absence of binding with nearest neighbor, correlation with RNA, etc) has been performed for ~70% of the assay.

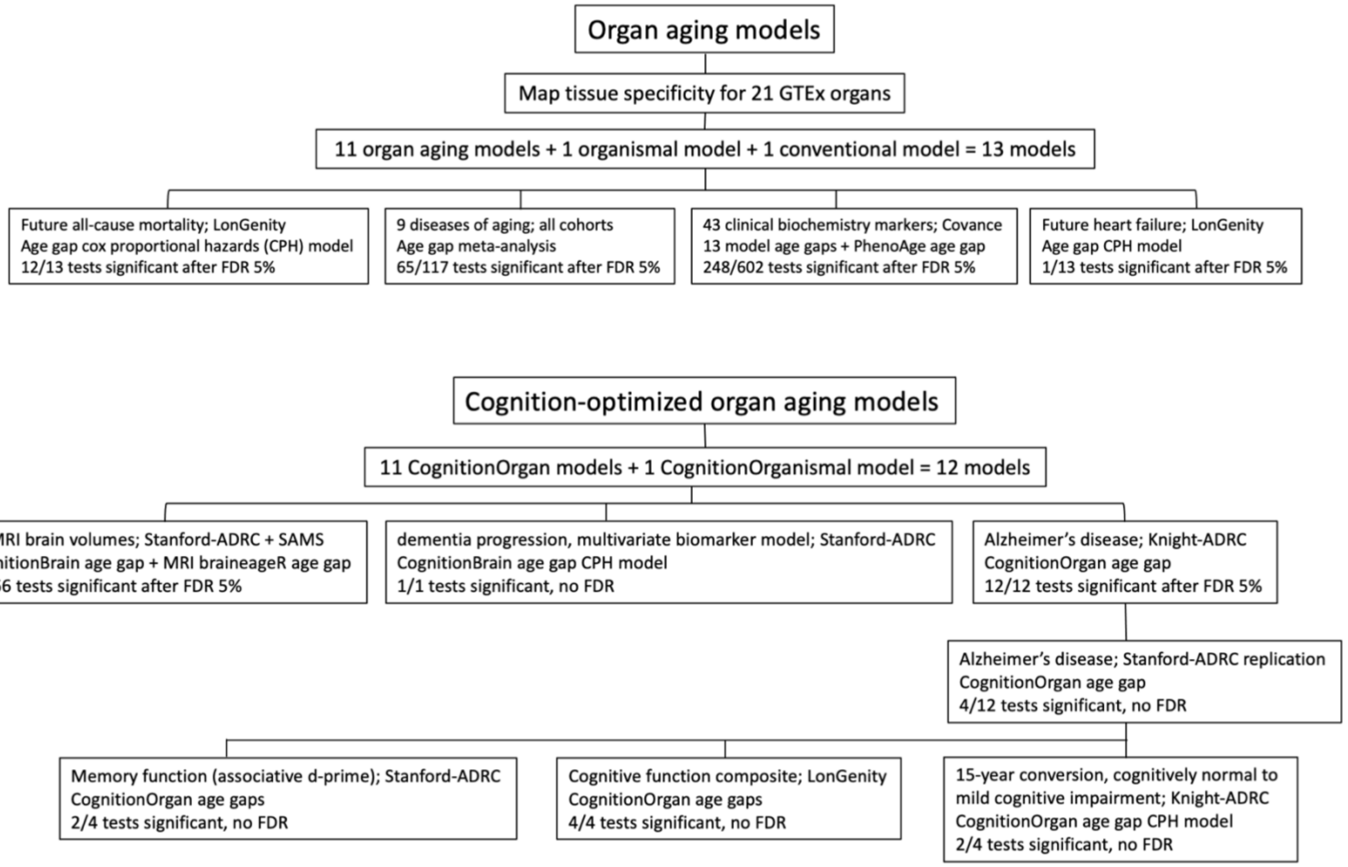

Supplementary Figure 2. Study design

a, Flow chart of our study design detailing all statistical tests done in the study.

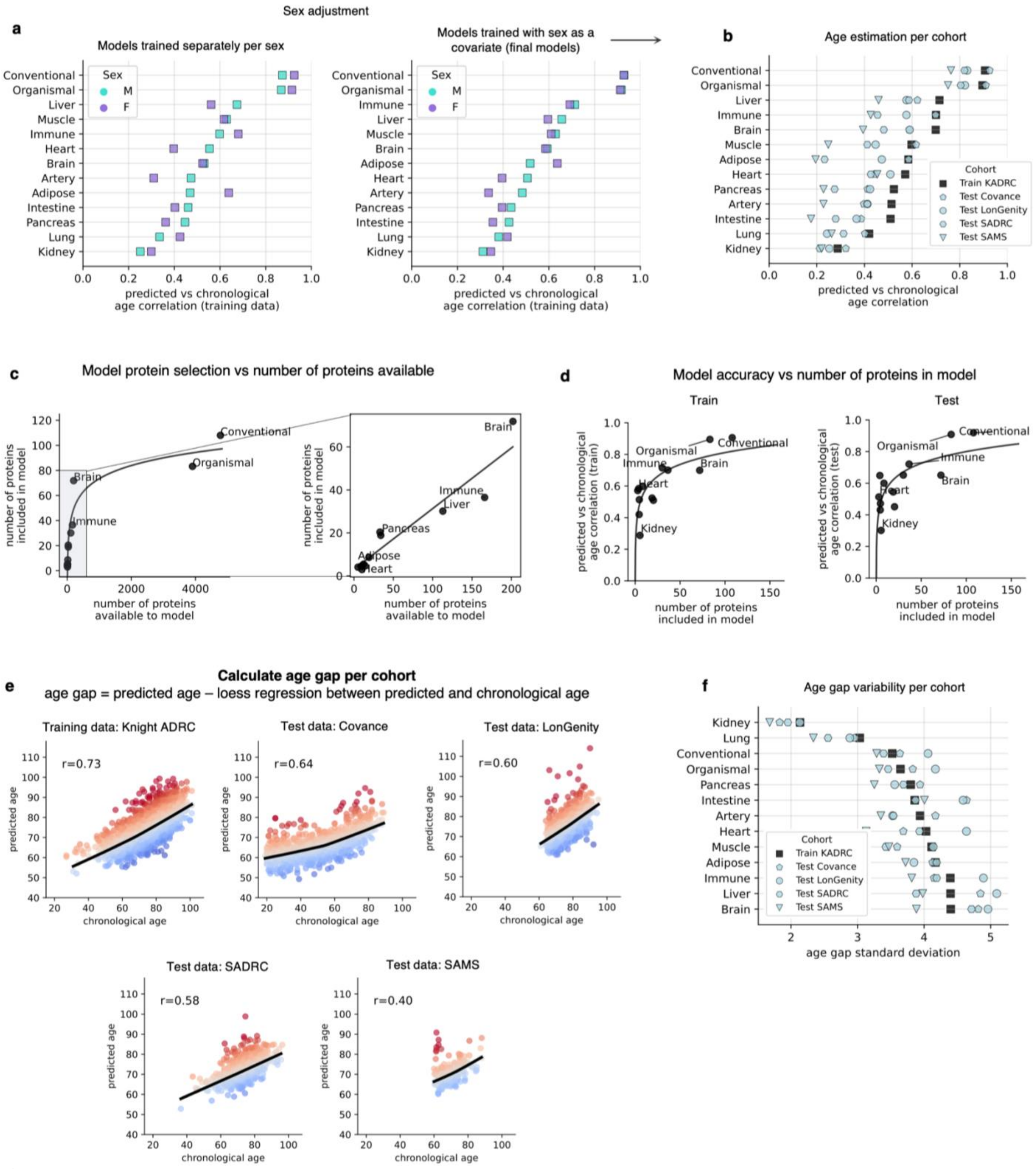

Supplementary Figure 3. Aging model characteristics and age gap calculation

a, Correlations between predicted vs chronological age in healthy individuals in the training (Knight-ADRC) cohorts for aging models trained separately per sex or trained with sex as a covariate. Models with sex as a covariate were used for all downstream analyses due to their performance, to extend the generality of the findings, and to reduce analytic complexity.

b, Correlations between predicted vs chronological age in healthy individuals in the training (Knight-ADRC) and test (Covance, LonGenity, Stanford-ADRC, SAMS) cohorts for all aging models. All aging models significantly estimated age across five independent cohorts.

c, Display of the relationship between the number of proteins available for model training and the average number of proteins selected by the bootstrapped models.

d, Display of the relationship between the average number of proteins selected by the bootstrapped models and the model accuracy in the train and test cohorts.

e, Calculation of organ age gaps per cohort. An individual's age gap is defined as the difference between the individual's predicted age and the lowess regression curve between predicted and chronological age.

f, Standard deviations of organ age gaps per cohort. Age gaps were z-score normalized separately per aging model for all downstream analyses to account for differences in model error and cohort effects.

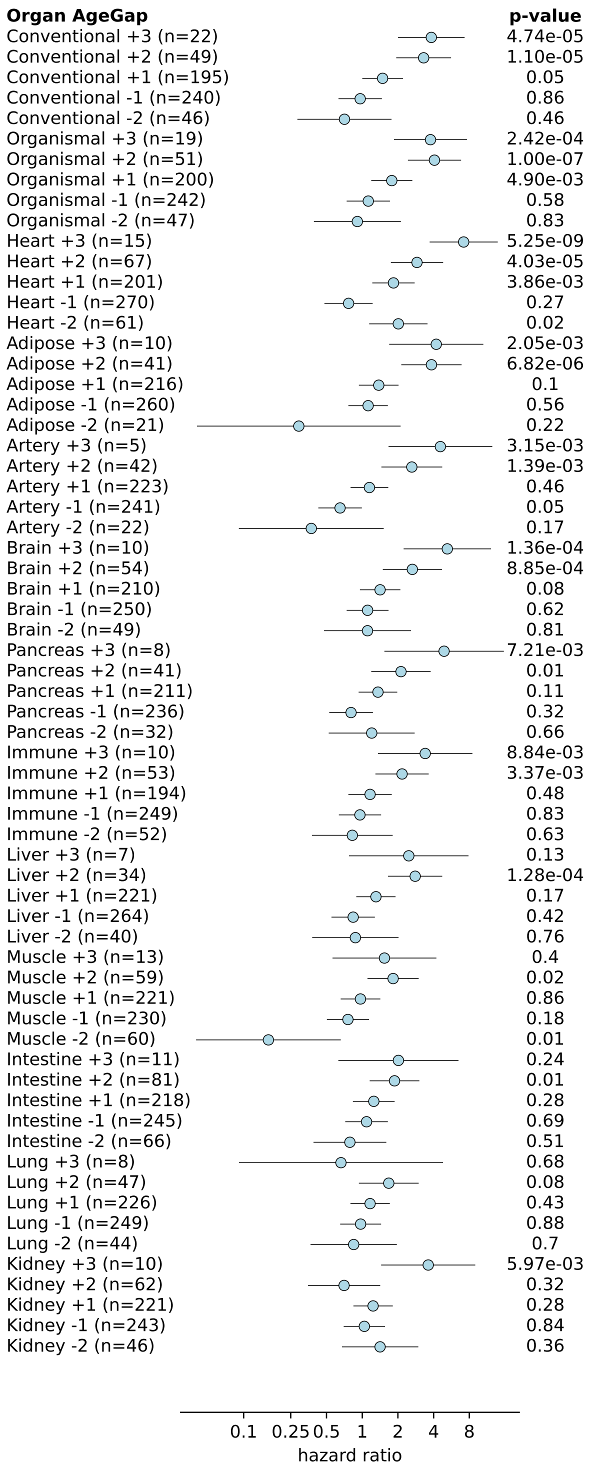

Supplementary Figure 4. Age gaps versus mortality risk, stratified by age gap bins.

a, Binned cox proportional hazard regression analysis in mortality risk, within 15 years in the LonGenity cohort (n=173 events out of 864 individuals). Individuals were grouped into different z-scored age gap bins: –2, –1, 0, +1, +2, +3 (–3 was removed due to low sample size). Bin limits were +/– 0.5. Each non-zero

group was compared with the zero group (denoting the non-zero group as 1 and the zero group as 0) for changes in mortality risk: MortalityRisk ~ *AgeGapBin (binary)* + Age + Sex. This analysis was performed for each aging model separately. Hazard ratios, 95% confidence intervals, p-values, and sample size for age gap bins are shown.

All error bars represent 95% confidence intervals.

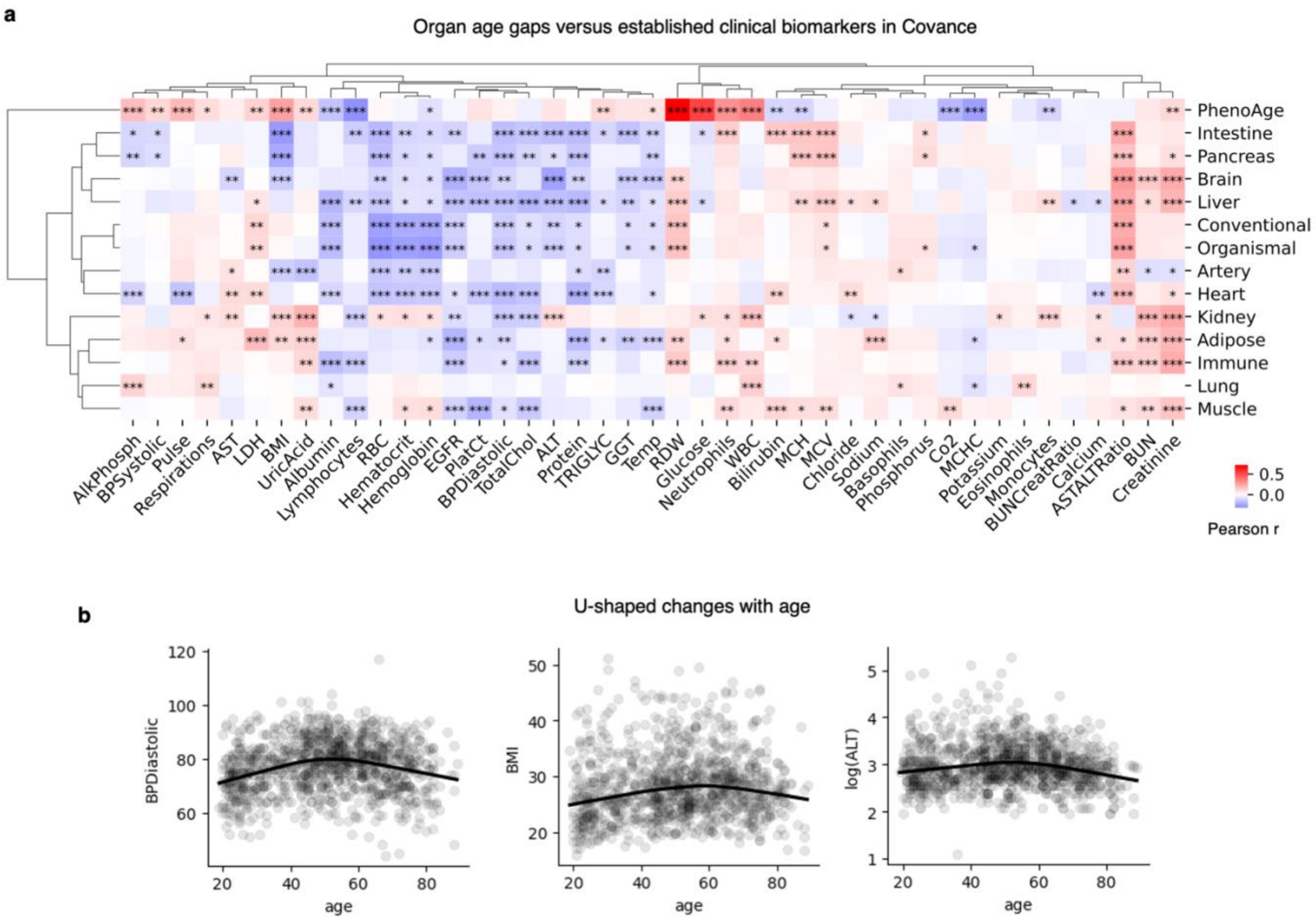

Supplementary Figure 5. Age gaps versus established clinical markers of aging, health, and disease.

a, Organ age gaps and the PhenoAge age gap were associated with 43 individual clinical markers of health and disease, controlling for age and sex (AgeGap ~ *Phenotype* + Age + Sex) in the Covance cohort. Phenotype covariate effect sizes and significance based on Benjami Hochberg correction for all associations are shown. Asterisks represent q-value thresholds: *q < 0.05; **q < 0.01; ***q < 0.001.

b, U-shaped relationship between age and certain traits, including diastolic blood pressure, BMI, and alanine transaminase are shown.

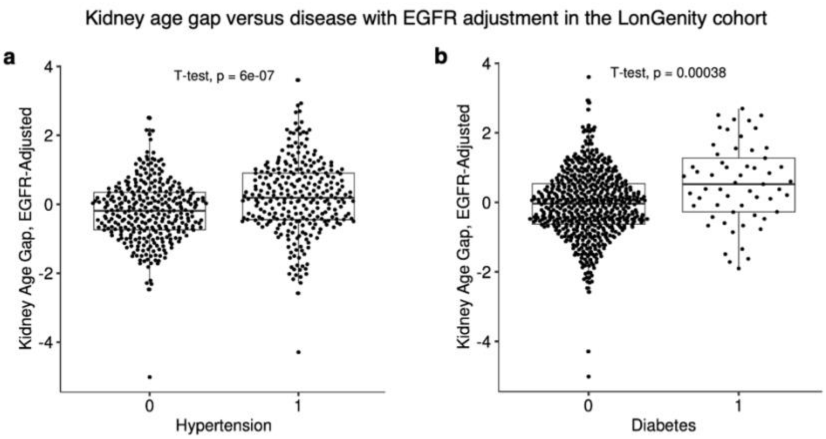

Supplementary Figure 6. Estimated glomerular filtration rate (EGFR) adjusted associations with disease.

a, Kidney age gap associations with hypertension, adjusted for EGFR in the LonGenity cohort (with hypertension n=280, without n=322). Two-tailed t-test used.

b, Kidney age gap associations with diabetes, adjusted for EGFR in the LonGenity cohort (with diabetes n=57, without n=581). Two-tailed t-test used.

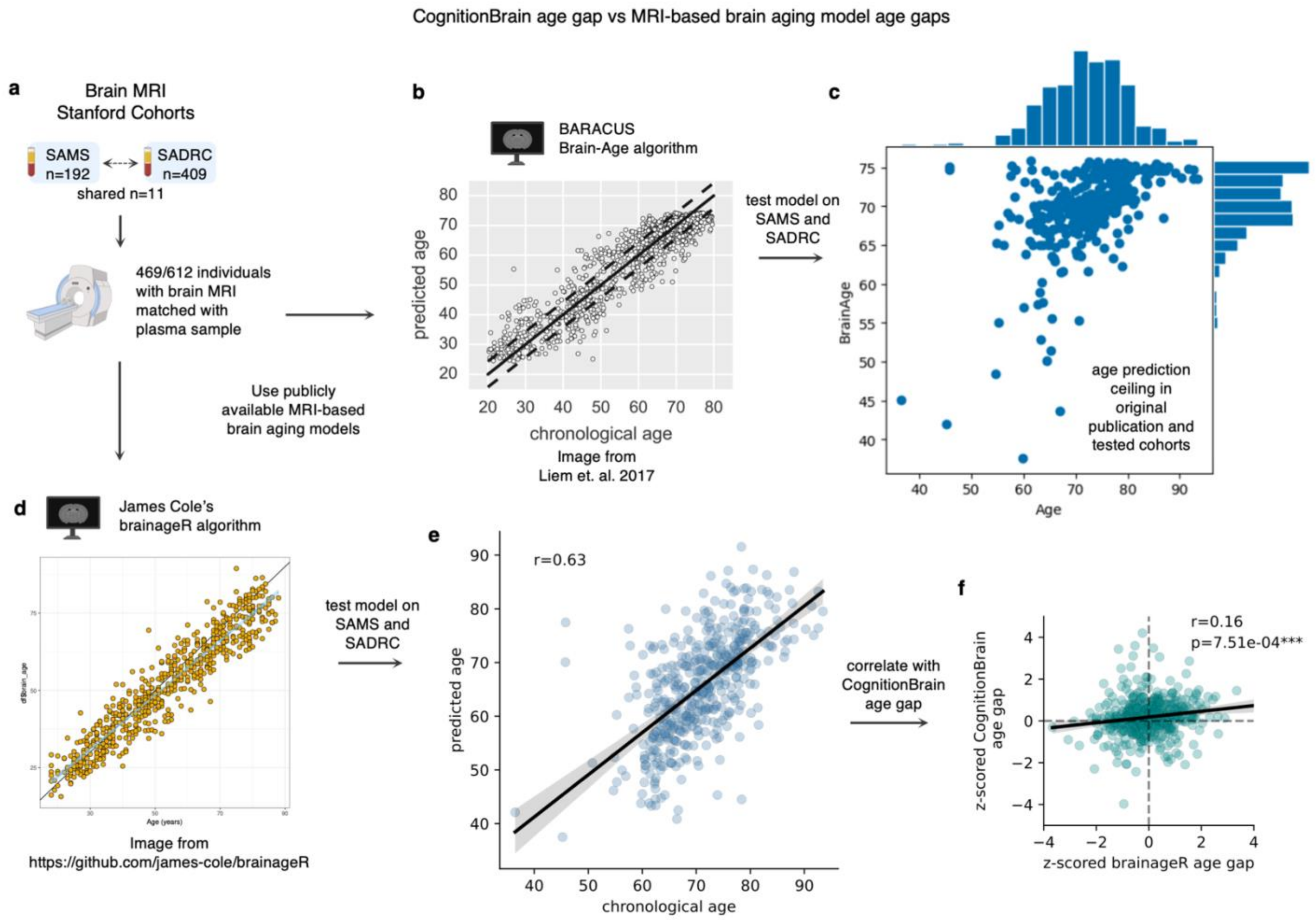

Supplementary Figure 7

a, CognitionBrain age gaps were associated with brain MRI volume in the Stanford-ADRC and SAMS cohorts (n=469).

b, The publicly available brain MRI based aging model, BARACUS Brain-Age, was tested in the Stanford-ADRC and SAMS cohorts. The age prediction image from the original publication Liem et. al. 2017 is shown. An age prediction ceiling can be observed.

c, The age prediction using BARACUS in the Stanford-ADRC and SAMS cohorts is shown. An age prediction ceiling can be observed in both the original publication and when tested in Stanford cohorts.

d, The publicly available brain MRI based aging model, brainageR, was tested in the Stanford-ADRC and SAMS cohorts. The age prediction image from the github is shown. No age prediction ceiling can be observed.

e, The age prediction using brainageR in the Stanford-ADRC and SAMS cohorts is shown. No age prediction ceiling can be observed.

f, The correlation between the CognitionBrain age gap and brainageR age gap was assessed and is shown.

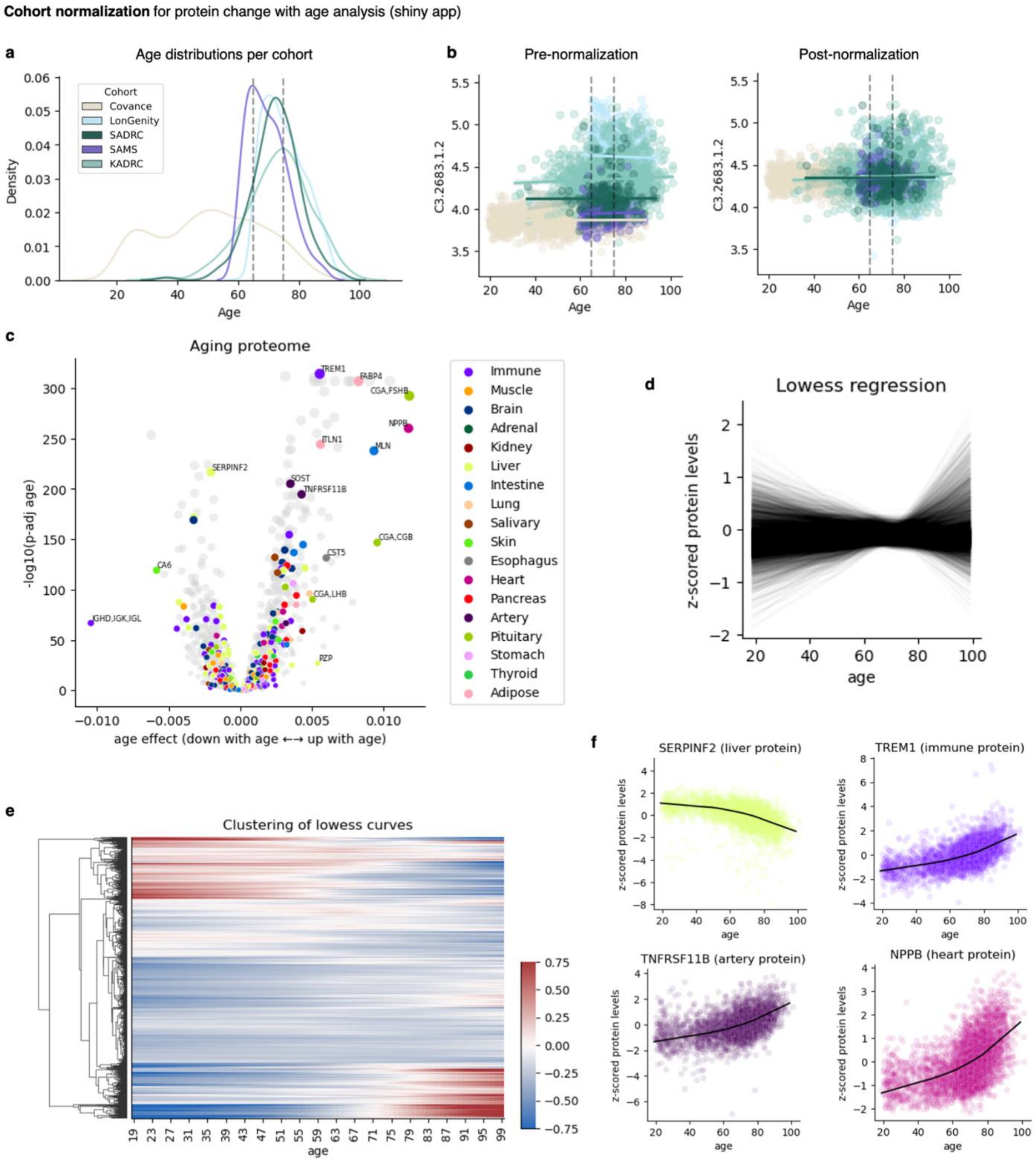

Supplementary Figure 8. Linear and non-linear changes with age in the plasma proteome.

a, Age distributions per cohort.

b, Cohort normalization to assess plasma proteome changes with age, independent of cohort age distribution. Cohort normalization was not applied for training and assessing aging models across cohorts.

c, Change with age in the plasma proteome (Protein ~ *Age* + Sex). Age effects and transformed Benjamini Hochberg adjusted p-values shown.

d, Locally weighted scatterplot smoothing (LOWESS) curves for aging plasma proteome shown.

e, LOWESS curves plotted as heatmap

f, Example proteins that change non-linearly with age.
